# Elevated Serum Vitamin B12 Levels as a Potential Biomarker for Solid Tumors in Jordanian Patients: A Retrospective Case–Control Study

**DOI:** 10.1155/ijbc/7833513

**Published:** 2026-03-06

**Authors:** Sahar Kamal Otoom, Lobna Gharaibeh, Anas Abed, Ibrahim Aldeeb

**Affiliations:** ^1^ Biopharmaceutics and Clinical Pharmacy Department, Faculty of Pharmacy, Al-Ahliyya Amman University, Amman, Jordan, ammanu.edu.jo; ^2^ Department of Pharmacy, Faculty of Pharmacy, Amman Arab University, Amman, Jordan, aau.edu.jo; ^3^ Department of Pharmacy, Faculty of Health Sciences, American University of Madaba, Madaba, Jordan, aum.edu.jo

**Keywords:** association, cancer, cross-sectional study, Jordan, prevalence, vitamin B12

## Abstract

**Background:**

Vitamin B12 deficiency is classically associated with anemia and neurological dysfunction. However, recent studies suggest that elevated plasma vitamin B12 may indicate increased short‐term cancer risk. This association remains largely unexplored in Middle Eastern populations, including Jordan, where cancer rates are rising and diagnosis often occurs at advanced stages due to limited screening.

**Objective:**

This study is aimed at investigating the association between serum vitamin B12 levels and the risk of colorectal, breast, and lung cancers in a Jordanian population, evaluating differences by cancer type and stage.

**Methods:**

A retrospective case–control study was conducted at King Abdullah University Hospital, Jordan, from January 2018 to December 2022. The study enrolled 260 patients diagnosed with colorectal, breast, or lung cancer and 260 matched healthy controls. Data collected included sociodemographic factors, clinical characteristics, and serum vitamin B12 levels.

**Results:**

Serum vitamin B12 levels were significantly higher in cancer patients compared with controls (579.23 ± 468.72 vs. 492.70 ± 174.36 pg/mL; *p* = 0.005). High vitamin B12 levels (> 800 pg/mL) occurred in 15.8% of cancer patients versus 1.5% of controls (*p* < 0.001). Vitamin B12 levels varied significantly by cancer type, being highest in lung cancer patients (669.53 ± 566.59 pg/mL) compared with breast (594.86 ± 468.9 pg/mL) and colorectal cancer patients (439.62 ± 291.89 pg/mL; *p* = 0.024). There was a strong positive correlation between vitamin B12 levels and cancer stage, peaking in Stage IV cancers, *r* = 0.629, *p* = 0.001.

**Conclusion:**

Elevated serum vitamin B12 levels are significantly associated with solid cancers in Jordanian patients, particularly pronounced in lung cancer and advanced stages. These findings do not imply a causal relationship, but rather suggest that serum vitamin B12 may function as a potential biomarker for cancer detection and disease monitoring in resource‐limited settings.

## 1. Introduction

Cancer remains a major global health challenge, contributing significantly to morbidity and mortality across diverse populations. In Jordan, cancer is the second leading cause of death after cardiovascular diseases, posing a substantial burden on the healthcare system [1]. According to the Jordan Cancer Registry, breast cancer is the most prevalent malignancy, accounting for 39.4% of cancers among women and 20.6% of all cancers in both sexes, followed by colorectal and lung cancers, which are among the top cancers affecting both men and women [2]. The incidence of these cancers has risen over the past decade, highlighting the urgent need to identify risk factors and biomarkers to enhance early detection and improve outcomes in Jordan′s resource‐constrained healthcare environment.

Vitamin B12, or cobalamin, is an essential water‐soluble vitamin critical for DNA synthesis, red blood cell production, and neurological function [3]. Vitamin B12 deficiency is widely recognized for its association with anemia and neurological disorders [4]. However, recent studies have highlighted a potential concern regarding high‐plasma B12 levels, which have been linked to an elevated short‐term risk of cancer, particularly in patients with solid tumors such as those of the lung, colorectal, and breast cancers [5]. A 2019 cohort study in the United Kingdom found that individuals with elevated B12 levels (> 600 pmol/L) had a higher 1‐year cancer risk compared with those with normal levels, especially for smoking‐ and hematological‐related cancers [6]. Similarly, a 2021 study reported that persistent elevation of plasma B12 was strongly associated with solid cancers, suggesting its potential as a biomarker [7]. However, findings are mixed, with some studies indicating no causal link or even low B12 levels in certain cancers, underscoring the need for further investigation to clarify these associations.

In Jordan, where late‐stage cancer diagnoses are common due to limited screening programs, identifying noninvasive biomarkers like B12 could significantly improve early detection and disease monitoring. Despite the growing body of evidence, few studies have explored this association in Middle Eastern populations, and none have specifically addressed the Jordanian context, where dietary patterns, genetic factors, and healthcare access may influence B12 levels and cancer risk.

This study addresses this gap by conducting a retrospective case–control study at King Abdullah University Hospital (KAUH) from 2018 to 2022 to investigate the association between serum vitamin B12 levels and the risk of colorectal, breast, and lung cancers in a Jordanian population. The objectives were to compare B12 levels between cancer patients and healthy controls, evaluate variations by cancer type and stage, and assess associations with clinical characteristics. By exploring whether elevated B12 levels can serve as a biomarker for cancer risk or progression, this research is aimed at contributing to improved cancer care strategies in Jordan and informing future studies in similar settings.

## 2. Methods

This retrospective case–control study was conducted at KAUH in Jordan, a tertiary care center, from January 2018 to December 2022. The study is aimed at investigating the association between serum vitamin B12 levels and cancer risk among patients with colorectal, breast, or lung cancer, the most prevalent cancers in Jordan. Ethical approval was obtained from the KAUH Institutional Review Board (IRB Number 2017/123), and the study adhered to the Declaration of Helsinki. Informed consent was waived due to the retrospective design, as data were extracted from existing medical records.

We enrolled 260 cancer patients (cases) diagnosed with colorectal, breast, or lung cancer, identified through KAUH medical records. Inclusion criteria for cases included a histopathologically confirmed cancer diagnosis and age between 18 and 80 years. Exclusion criteria included diagnoses of other cancer types or incomplete medical records. Controls consisted of 260 age‐ and gender‐matched healthy individuals recruited from KAUH outpatient clinics during the same period. Controls had no history of cancer or chronic diseases and were not taking B12 supplements.

Data were extracted from medical records, including sociodemographic variables (age, gender, BMI, and smoking status), clinical variables (cancer type, stage, treatment type, duration of cancer, and death state), and serum B12 levels. Cancer stage was classified using the TNM system (Stages I–IV). Smoking status was categorized as never, former, or current. BMI was calculated as weight (kg)/height (m^2^) and categorized as underweight (< 18.5), normal (18.5–24.9), overweight (25–29.9), or obese (≥ 30). All data were de‐identified to ensure patient confidentiality.

Descriptive statistics were reported as means ± standard deviations (SDs) for continuous variables (e.g., age and B12 levels) and counts/percentages for categorical variables (e.g., gender and smoking status). Independent samples *t*‐test was used to compare continuous variables between cases and controls. Chi‐squared tests assessed differences in categorical variables. One‐way ANOVA with post hoc Scheffe were used to compare continuous variables between more than two categories. Pearson correlation analysis examined the relationship between two sets of continuous data. A *p* value < 0.05 was considered statistically significant. All analyses were performed using SPSS Version 25 (IBM Corp., Armonk, New York, United States).

## 3. Results

Two hundred sixty patients with cancer were matched with 260 individuals with no cancer diagnosis. The sociodemographic and clinical characteristics of the participants were presented in Table 1.

**Table 1:** Sociodemographic and clinical characteristics of participants.

Characteristic	Cases (*n* = 260)	Controls (*n* = 260)	*p*
Age (years, mean ± SD)	57.32 ± 9.8	58.74 ± 9.79	0.102
Gender, *n* (%)			0.302
Female	171 (65.8%)	182 (70.0%)	
Male	89 (34.2%)	78 (30.0%)	
BMI (mean ± SD)	23.64 ± 3.23	22.57 ± 2.62	0.060
BMI categories, *n* (%)			0.001 ^∗^
Underweight	10 (3.8%)	0 (0.0%)	
Normal weight	190 (73.1%)	224 (86.2%)	
Overweight	53 (20.4%)	33 (12.7%)	
Obese	7 (2.7%)	3 (1.2%)	
Smoking, *n* (%)			0.441
Never	112 (43.1%)	109 (41.9%)	
Former	99 (38.1%)	111 (42.7%)	
Current	49 (18.8%)	40 (15.4%)	
Cancer type, *n* (%)			—
Bronchus or lung	62 (23.8%)	—	
Breast	142 (54.8%)	—	
Colon	56 (21.5%)	—	
Stage of cancer, *n* (%)			—
Stage I	78 (30.0%)	—	
Stage II	91 (35.0%)	—	
Stage III	44 (16.9%)	—	
Stage IV	47 (18.1%)	—	
Treatment for cancer, *n* (%)			—
Surgical excision	5 (1.9%)	—	
Chemotherapy	109 (41.9%)	—	
Radiotherapy	99 (38.1%)	—	
Combined therapy	47 (18.1%)	—	
Time of B12 measurement, *n* (%)			—
Before diagnosis	90 (34.6%)	—	
After diagnosis	170 (65.4%)	—	
Death state, *n* (%)			—
Alive	156 (60.0%)	—	
Dead	104 (40.0%)	—	

*Note:* *p* values from independent *t*‐tests (continuous variables) or chi‐squared tests (categorical variables). Data sourced from medical records.

^∗^ *p* < 0.05, statistically significant.

Laboratory parameters revealed significant differences between cases and controls. All controls had normal liver and renal function tests and normal hemoglobin levels, Table 2.

**Table 2:** Laboratory parameters of cases and controls.

Variable	Cases (*N* = 260)	Controls (*N* = 260)	*p*
CBC, *n* (%)			0.001 ^∗^
Normal	161 (61.9%)	260 (100%)	
Abnormal	99 (38.1%)	0 (0%)	
Liver function test, *n* (%)			0.001 ^∗^
Normal	132 (50.8%)	260 (100%)	
Abnormal	128 (49.2%)	0 (0%)	
Renal function test, *n* (%)			0.001 ^∗^
Normal	191 (73.5%)	260 (100%)	
Abnormal	69 (26.5%)	0 (0%)	
Hemoglobin (mg/dL, mean ± SD)	11.3 ± 2.2	13.5 ± 1.2	0.001 ^∗^
Mean corpuscular volume (fL, mean ± SD)	103.3 ± 21.3	89.5 ± 6.45	0.001 ^∗^
ALT (U/L, mean ± SD)	71.6 ± 48.9	24.5 ± 5.2	0.001 ^∗^
AST (U/L, mean ± SD)	68.6 ± 48.9	17.6 ± 3.8	0.001 ^∗^
Urea (mg/dL, mean ± SD)	29.2 ± 20.3	20.2 ± 3.7	0.001 ^∗^
Creatinine (mg/dL, mean ± SD)	2.06 ± 2.17	0.90 ± 0.23	0.001 ^∗^

*Note:* *p* values from independent *t*‐tests (continuous variables) or chi‐squared tests (categorical variables). Data sourced from medical records.

^∗^ *p* < 0.05, statistically significant.

Serum vitamin B12 levels were significantly higher in cancer patients compared with healthy controls, as shown in Table 3. The mean B12 level in cases was 579.23 ± 468.72 pg/mL, compared with 492.70 ± 174.36 pg/mL in controls; despite large standardized deviations, the *p* value indicates significant differences since overlap of confidence intervals does not invalidate statistical significance (*p* = 0.005) [8]. When classified into categories, 15.8% of cases (*n* = 41) had high B12 levels (> 800 pg/mL) compared with only 1.5% of controls (*n* = 4; *p* < 0.001), highlighting a marked prevalence of elevated B12 in cancer patients.

**Table 3:** Serum vitamin B12 levels in cases and controls.

Measure	Cases (*n* = 260)	Controls (*n* = 260)	*p*
	**Continuous data**		
B12 level (pg/mL, mean ± SD)	579.23 ± 468.72	492.70 ± 174.36	0.005 ^∗^
	**Categorical data**		
B12 categories, *n* (%)			< 0.001 ^∗^
Low/normal (200–800 pg/mL)	219 (84.2%)	256 (98.5%)	
High (> 800 pg/mL)	41 (15.8%)	4 (1.5%)	

*Note:* *p* values from independent *t*‐tests (continuous variables) or chi‐squared tests (categorical variables). Data sourced from medical records.

^∗^ *p* < 0.05, statistically significant.

Vitamin B12 levels varied significantly across cancer types. Among the 260 cancer patients, those with bronchus or lung cancer exhibited the highest mean B12 level at 669.53 ± 566.59 pg/mL, Table 4.

**Table 4:** Vitamin B12 levels by cancer type.

Cancer type	*n*	B12 level (pg/mL, *m* *e* *a* *n* ± *S* *D*)	*p* (ANOVA)	Post hoc Scheffe *p*
Bronchus/lung	62	669.53 ± 566.59	0.024 ^∗^	Lung vs. colon: 0.028 ^∗^
Breast	142	594.86 ± 468.9		Lung vs. breast: 0.572
Colon	56	439.62 ± 291.89		Breast vs. colon: 0.107

*Note:* ANOVA and post hoc Scheffe tests used. Data sourced from medical records.

^∗^ *p* < 0.05, statistically significant.

A strong positive correlation (*r* = 0.629, *p* = 0.001) was observed between B12 levels and cancer stage, indicating that B12 rises as cancer progresses. Additionally, vitamin B12 levels increased significantly with cancer stage. Among the 260 cancer patients, mean B12 levels were lowest in Stage I and highest in Stage IV, Table 5.

**Table 5:** Vitamin B12 levels by cancer stage.

Cancer stage	*n*	B12 level (pg/mL, *m* *e* *a* *n* ± *S* *D*)	*p* (ANOVA)	Correlation factor
Stage I	78	347.00 ± 178.43	< 0.001 ^∗^	0.629
Stage II	91	452.90 ± 232.81		
Stage III	44	480.53 ± 293.30		
Stage IV	47	1301.60 ± 575.9		

*Note:* ANOVA used for group comparison; correlation factor indicates the relationship between B12 levels and cancer stage.

^∗^ *p* < 0.05, statistically significant.

## 4. Discussion

Our retrospective case–control study demonstrates that serum vitamin B12 levels are significantly elevated in cancer patients compared with healthy controls. Patients with solid tumors (colorectal, breast, and lung) had higher B12 concentrations than age‐ and sex‐matched cancer‐free individuals, consistent with reports that elevated plasma B12 is associated with malignancies [7]. Notably, lung cancer patients exhibited the highest B12 levels (mean ~670 pg/mL), significantly greater than those with colorectal cancer, and B12 rose progressively with advancing stage—from a mean ~347 pg/mL in Stage I to ~1302 pg/mL in Stage IV. This strong positive correlation between B12 and cancer stage suggests that higher cobalamin levels reflect greater tumor burden or extent of disease. Indeed, prior studies have observed that B12 elevations tend to increase in metastatic or advanced cancer, and extremely high B12 has been linked to worse prognosis (e.g. 1‐year survival as low as 36% when B12 > 800 pmol/L) [9]. Our findings align with this pattern and support the notion that cobalamin levels may serve as a surrogate marker of disease extent in cancer patients.

The biological mechanisms underlying cancer‐related B12 elevation are multifactorial. One proposed mechanism is altered cobalamin transport due to increased production of B12‐binding proteins (haptocorrin/transcobalamin I) by tumor and inflammatory cells [5, 10, 11]. Malignant cells and activated neutrophils can release haptocorrin, which binds cobalamin and raises total serum B12 levels without reflecting functional B12 utilization [12]. In addition, liver dysfunction is a well‐recognized contributor to hypercobalaminemia in cancer [13]. Many advanced cancers (particularly metastases to the liver) disrupt normal hepatic storage and clearance of B12, causing a spillover of cobalamin into the circulation [14]. Altered cobalamin metabolism in malignancy may also play a role; rapid cell turnover could increase B12 demand, yet if cancer or associated inflammation saturates the uptake pathway, excess B12 may accumulate in blood. The fact that B12 elevations in our cohort were largely independent of gender, body mass index, or smoking status further suggests a tumor‐driven phenomenon. We observed neither significant differences in B12 levels between male and female patients nor across smoking categories (never, former, and current), which is in line with a general population study in Jordan that found no sex difference in B12 status [15]. Even smoking—which has been associated with slightly higher B12 levels in healthy Jordanians [16]—did not influence B12 in our cancer patients, indicating that the presence and severity of cancer outweigh lifestyle factors in determining cobalamin levels.

Our results are contextualized by growing literature on vitamin B12 as a potential cancer biomarker. In Western populations, elevated B12 has been linked to increased cancer incidence and mortality. For example, a large Danish cohort first reported that patients with supranormal B12 had a higher likelihood of being diagnosed with cancer in the following year [17]. Similarly, a United Kingdom primary care study found that individuals with B12 > 600 pmol/L had a 1.7–4.7 fold higher 1‐year risk of cancer (especially smoking‐related and hematological cancers) compared with those with normal levels [6]. More recently, persistent B12 elevation has emerged as a strong red flag. Lacombe et al. (2021) showed that patients whose B12 remained ≥ 1000 ng/L on repeat testing had nearly a sixfold greater hazard of developing solid cancers relative to those with normal B12 [7]. Our finding that many cancer patients (particularly in late stages) have B12 exceeding this threshold reinforces the relevance of these observations to clinical practice. At the same time, some studies have reported null or conflicting results. A meta‐analysis of one‐carbon metabolism biomarkers, for instance, found no significant overall difference in serum B12 between lung cancer cases and controls [18]. Additionally, a Mendelian randomization analysis concluded that high B12 status is likely a consequence of cancer rather than a direct cause, at least in lung cancer [19]. These discrepancies highlight that elevated B12 is a marker of underlying pathology, not a causal risk factor per se. Our study supports this interpretation where the excess B12 in patients is most plausibly due to the effect of cancer on the body rather than pre‐existing high B12 causing cancer.

Importantly, our work addresses a gap in data from the Middle East. Few studies in the region have examined vitamin B12 in relation to cancer, and to our knowledge, this is the first study from Jordan to evaluate this association. The Jordanian population has distinct nutritional patterns and a high prevalence of B12 deficiency in the general population (approximately one‐third of adults) [15]. Previous research in Jordan regarding B12 has mostly focused on deficiencies or nonmalignant conditions where several studies reported widespread low B12 status [16, 20]. Thus, observing abnormally high B12 levels in cancer patients is striking and underlines a pathological deviation from typical levels. Therefore, our findings provide novel evidence that elevated B12 could serve as a biomarker of cancer in Jordanian patients, an insight not previously documented.

From a clinical standpoint, these findings carry several implications. First, serum B12 could be a useful adjunct for early cancer detection or alerting physicians to occult malignancy. Patients who present with unexplained hypercobalaminemia (and no history of B12 supplementation or myeloproliferative disorder) should prompt clinicians to consider a thorough evaluation for hidden cancers. Second, B12 levels may have a prognostic value. The marked escalation of B12 with advanced stage implies that very high levels could signal more aggressive or disseminated disease. Monitoring B12 in diagnosed cancer patients might help identify progression or relapse. Notably, one longitudinal study found that B12 levels decreased by ~172 ng/L per month in patients receiving curative treatment, whereas they continued to rise (~157 ng/L per month) in those on supportive care with uncontrolled cancer [21]. This dynamic change suggests that falling B12 could correlate with treatment response, whereas a persistently rising B12 might indicate treatment failure or tumor growth. Such trends position B12 as a potential disease monitoring tool alongside other markers. In resource‐limited settings, the availability of a cheap, routine test that reflects tumor status is attractive. However, we caution that B12 is a nonspecific marker—elevated levels can occur in various conditions such as liver cirrhosis, renal impairment, and inflammation [22]. Therefore, an isolated high B12 should be interpreted in context and confirmed persistent before initiating extensive cancer investigations. The study of vitamin B12 as a member of the huge available data that includes lab tests and genetics represents a small piece of the puzzle. Studies using artificial intelligence models for the detection and diagnosis of cancer have proven successful and may provide a more advanced and comprehensive utilization of data [23].

This study has some limitations. The retrospective design inherently limits the ability to infer causality or temporal relationships. Additionally, the single‐center Jordanian sample may limit generalizability, although our results mirror those seen internationally. Another limitation is that although we excluded patients on B12 supplements and matched controls to cases, unmeasured confounders (such as dietary B12 intake) could not be accounted for. Moreover, patients with cancer had abnormal liver and renal function tests, which might influence vitamin B12 levels via several mechanisms. Consequently, underlying hepatic or renal dysfunction may contribute to high vitamin B12 levels.

Additionally, the study lacks mechanistic measurements, therefore, causal or mechanistic conclusions cannot be confirmed. This limitation provides insights into possible future studies that examine these mechanistic pathways. Despite these limitations, the consistency of our findings with biological expectations and prior studies lends credibility to the association between high B12 and cancer.

Future prospective studies are needed to evaluate the utility of vitamin B12 as a predictive biomarker for cancer in our region. It would also be valuable to combine B12 with other biomarkers—for instance, inflammatory markers (C‐reactive protein), liver enzymes, or classic tumor markers—to improve diagnostic accuracy. Since B12 elevation is not cancer‐specific, a multimarker panel might better discriminate cancer‐related B12 rises from benign causes. In addition, interventional studies might explore whether B12 normalization has any impact on outcomes, though B12 is more likely a consequence than a driver of cancer. Another promising avenue is to monitor B12 trends during cancer therapy.

## 5. Conclusion

In conclusion, our study is the first from Jordan to report that cancer patients have significantly higher vitamin B12 levels than healthy individuals, with particularly pronounced elevations in lung cancer and late‐stage disease. These results strengthen the evidence that elevated B12 is not merely a potential biochemical anomaly but a potential red flag for underlying cancer, likely reflecting tumor‐induced alterations in cobalamin metabolism. Ultimately, this research contributes a novel regional perspective and suggests that a vitamin commonly associated with nutritional status might also serve as a meaningful biomarker in the oncology field, aiding in the fight against cancer in Jordan and beyond.

## Author Contributions

Conceptualization: L.G. and I.A.; methodology: L.G. and I.A.; data: L.G. and S.K.O.; analysis: L.G. and S.K.O.; writing: A.A., L.G., and S.K.O.; editing: L.G., A.A., S.K.O., and I.A.

## Funding

No funding was received for this manuscript.

## Conflicts of Interest

The authors declare no conflicts of interest.

## Data Availability

The data that support the findings of this study are available on request from the corresponding author. The data are not publicly available due to privacy or ethical restrictions.

## References

[1] Abdel-Razeq H. , Al-Ibraheem A. , Al-Rabi K. , Shamiah O. , Al-Husaini M. , and Mansour A. , Cancer Care in Resource-Limited Countries: Jordan as an Example, JCO Global Oncology. (2024) 10, e2400237, 10.1200/GO.24.00237, 39361906. 39361906

[2] Abdel-Razeq H. , Mansour A. , and Jaddan D. , Breast Cancer Care in Jordan, JCO Global Oncology. (2020) 6, 260–268, 10.1200/JGO.19.00279, 32083950. 32083950 PMC7051801

[3] Aburayyan W. , Zakaraya Z. , Hamad M. , Majali I. S. , Abu Dayyih W. , Seder N. , Alkhadeir H. , and Khaleel A. , Improving HbA1c Levels by Methylcobalamin Vitamin in Diabetic Volunteers, Combined With Dapagliflozin as Type 2 Diabetes Mellitus Routine Treatment: A Controlled Randomized, Double-Blind Trial, Iranian Journal of Medical Sciences. (2025) 50, no. 5, 324–333, 10.30476/ijms.2024.101606.3423, 40433181. 40433181 PMC12104543

[4] Jajoo S. S. , Zamwar U. M. , and Nagrale P. , Etiology, Clinical Manifestations, Diagnosis, and Treatment of Cobalamin (Vitamin B12) Deficiency, Cureus. (2024) 16, no. 1, e52153, 10.7759/cureus.52153, 38344487. 38344487 PMC10859001

[5] Arendt J. F. H. , Farkas D. K. , Pedersen L. , Nexo E. , and Sørensen H. T. , Elevated Plasma Vitamin B12 Levels and Cancer Prognosis: A Population-Based Cohort Study, Cancer Epidemiology. (2016) 40, 158–165, 10.1016/j.canep.2015.12.007, 2-s2.0-84960089929, 26724465. 26724465

[6] Arendt J. F. H. , Sørensen H. T. , Horsfall L. J. , and Petersen I. , Elevated Vitamin B12 Levels and Cancer Risk in UK Primary Care: A THIN Database Cohort Study, Cancer Epidemiology, Biomarkers & Prevention. (2019) 28, no. 4, 814–821, 10.1158/1055-9965.EPI-17-1136, 2-s2.0-85063916683, 30642843. 30642843

[7] Lacombe V. , Chabrun F. , Lacout C. , Ghali A. , Capitain O. , Patsouris A. , Lavigne C. , and Urbanski G. , Persistent Elevation of Plasma Vitamin B12 is Strongly Associated With Solid Cancer, Scientific Reports. (2021) 11, no. 1, 13361, 10.1038/s41598-021-92945-y, 34172805. 34172805 PMC8233305

[8] Greenland S. , Senn S. J. , Rothman K. J. , Carlin J. B. , Poole C. , Goodman S. N. , and Altman D. G. , Statistical Tests, P Values, Confidence Intervals, and Power: A Guide to Misinterpretations, European Journal of Epidemiology. (2016) 31, no. 4, 337–350, 10.1007/s10654-016-0149-3, 2-s2.0-84971612122, 27209009. 27209009 PMC4877414

[9] Urbanski G. , Hamel J. F. , Prouveur B. , Annweiler C. , Ghali A. , Cassereau J. , Lozac′h P. , Lavigne C. , and Lacombe V. , Strength of the Association of Elevated Vitamin B12 and Solid Cancers: An Adjusted Case-Control Study, Journal of Clinical Medicine. (2020) 9, no. 2, 474, 10.3390/jcm9020474, 32050436. 32050436 PMC7073937

[10] Serraj K. , Mecili M. , Housni I. , and Andrès E. , Hypervitaminemia B12 (High Level of Cobalamin): Physiopathology, Role and Interest in Clinical Practice, Presse Médicale. (2011) 40, no. 12, pt. 1, 1120–1127, 10.1016/j.lpm.2011.08.010, 2-s2.0-84856197367, 22023830. 22023830

[11] Arendt J. F. B. and Nexo E. , Cobalamin Related Parameters and Disease Patterns in Patients With Increased Serum Cobalamin Levels, PLoS One. (2012) 7, no. 9, e45979, 10.1371/journal.pone.0045979, 2-s2.0-84866657517, 23029349. 23029349 PMC3448722

[12] Obeid R. , High Plasma Vitamin B12 and Cancer in Human Studies: A Scoping Review to Judge Causality and Alternative Explanations, Nutrients. (2022) 14, no. 21, 4476, 10.3390/nu14214476, 36364737. 36364737 PMC9658086

[13] Sugihara T. , Koda M. , Okamoto T. , Miyoshi K. , Matono T. , Oyama K. , Hosho K. , Okano J. I. , Isomoto H. , and Murawaki Y. , Falsely Elevated Serum Vitamin B(12) Levels Were Associated With the Severity and Prognosis of Chronic Viral Liver Disease, Yonago Acta Medica. (2017) 60, no. 1, 31–39, 28331419. 28331419 PMC5355842

[14] Andrès E. , Serraj K. , Zhu J. , and Vermorken A. J. M. , The Pathophysiology of Elevated Vitamin B12 in Clinical Practice, QJM: An International Journal of Medicine. (2013) 106, no. 6, 505–515, 10.1093/qjmed/hct051, 2-s2.0-84878631898, 23447660. 23447660

[15] El-Khateeb M. , Khader Y. , Batieha A. , Jaddou H. , Hyassat D. , Belbisi A. , and Ajlouni K. , Vitamin B12 Deficiency in Jordan: A Population-Based Study, Annals of Nutrition & Metabolism. (2014) 64, no. 2, 101–105, 10.1159/000355440, 2-s2.0-84902302472, 24943588. 24943588

[16] Abu-Shanab A. , Zihlif M. , Rbeihat M. N. , Shkoukani Z. W. , Khamis A. , Isleem U. , and Dardas L. A. , Vitamin B(12) Deficiency Among the Healthy Jordanian Adult Population: Diagnostic Levels, Symptomology and Risk Factors, Endocrine, Metabolic & Immune Disorders Drug Targets. (2021) 21, no. 6, 1107–1114, 10.2174/1871530320999200831230205, 32875992. 32875992

[17] Arendt J. F. B. , Pedersen L. , Nexo E. , and Sørensen H. T. , Elevated Plasma Vitamin B12 levels as a Marker for Cancer: A Population-Based Cohort Study, Journal of the National Cancer Institute. (2013) 105, no. 23, 1799–1805, 10.1093/jnci/djt315, 2-s2.0-84890544359, 24249744. 24249744 PMC3848986

[18] Yang J. , Li H. , Deng H. , and Wang Z. , Association of One-Carbon Metabolism-Related Vitamins (Folate, B6, B12), Homocysteine and Methionine With the Risk of Lung Cancer: Systematic Review and Meta-Analysis, Frontiers in Oncology. (2018) 8, 493, 10.3389/fonc.2018.00493, 2-s2.0-85063297795, 30430082. 30430082 PMC6220054

[19] Fanidi A. , Carreras-Torres R. , Larose T. L. , Yuan J. M. , Stevens V. L. , Weinstein S. J. , Albanes D. , Prentice R. , Pettinger M. , Cai Q. , Blot W. J. , Arslan A. A. , Zeleniuch-Jacquotte A. , McCullough M. , le Marchand L. , Wilkens L. R. , Haiman C. A. , Zhang X. , Stampfer M. J. , Smith-Warner S. A. , Giovannucci E. , Giles G. G. , Hodge A. M. , Severi G. , Johansson M. , Grankvist K. , Langhammer A. , Brumpton B. M. , Wang R. , Gao Y. T. , Ericson U. , Bojesen S. E. , Arnold S. M. , Koh W. P. , Shu X. O. , Xiang Y. B. , Li H. , Zheng W. , Lan Q. , Visvanathan K. , Hoffman-Bolton J. , Ueland P. M. , Midttun Ø. , Caporaso N. E. , Purdue M. , Freedman N. D. , Buring J. E. , Lee I. M. , Sesso H. D. , Michael Gaziano J. , Manjer J. , Relton C. L. , Hung R. J. , Amos C. I. , Johansson M. , Brennan P. , and LC3 consortium and the TRICL consortium , Is High Vitamin B12 Status a Cause of Lung Cancer?, International Journal of Cancer. (2019) 145, no. 6, 1499–1503, 10.1002/ijc.32033, 2-s2.0-85060173493, 30499135. 30499135 PMC6642017

[20] Farah H. S. , Hajleh M. A. , Shalan N. A. , AL ASSI G. A. , and Alqaisi T. A. , The Association Between the Levels of Ferritin, TSH, Zinc, Hb, Vitamin B12, Vitamin D and the Hair Loss Among Different Age Groups of Women: A Retrospective Study, International Journal of Pharmaceutical Research. (2021) 13, 143–148, 10.31838/ijpr/2021.13.02.038.

[21] Lacombe V. , Patsouris A. , Delattre E. , Lacout C. , and Urbanski G. , Evolution of Plasma Vitamin B(12) in Patients With Solid Cancers During Curative Versus Supportive Care, Archives of Medical Science. (2021) 17, no. 6, 1811–1815, 10.5114/aoms/140974, 34900064. 34900064 PMC8641499

[22] Argan O. , Ural D. , Karauzum K. , Bozyel S. , Aktas M. , Karauzum I. Y. , Kozdag G. , and Agacdiken Agir A. , Elevated Levels of Vitamin B12 in Chronic Stable Heart Failure: A Marker for Subclinical Liver Damage and Impaired Prognosis, Therapeutics and Clinical Risk Management. (2018) 14, 1067–1073, 10.2147/TCRM.S164200, 2-s2.0-85049843628, 29922067. 29922067 PMC5995286

[23] AlShourbaji I. , Kachare P. , Zogaan W. , Muhammad L. J. , and Abualigah L. , Learning Features Using an Optimized Artificial Neural Network for Breast Cancer Diagnosis, SN Computer Science. (2022) 3, 10.1007/s42979-022-01129-6.

